# tRNA-derived fragments as novel potential biomarkers for relapsed/refractory multiple myeloma

**DOI:** 10.1186/s12859-021-04167-8

**Published:** 2021-05-11

**Authors:** Cong Xu, Ting Liang, Fangrong Zhang, Jing Liu, Yunfeng Fu

**Affiliations:** 1 Department of Hematology, The Third Xiangya Hospital of Central South University, Changsha, 410000 China; 2 Department of Blood Transfusion, The Seventh Affiliated Hospital of Sun Yat-Sen University, Shenzhen, 518000 China; 3 Department of Blood Transfusion, The Third Xiangya Hospital of Central South University, Changsha, 410000 China

**Keywords:** Multiple myeloma, TRNA related fragments, TRNA halves, Relapsed/refractory, Biomarkers

## Abstract

**Background:**

tRNA-derived fragments have been reported to be key regulatory factors in human tumors. However, their roles in the progression of multiple myeloma remain unknown.

**Results:**

This study employed RNA-sequencing to explore the expression profiles of tRFs/tiRNAs in new diagnosed MM and relapsed/refractory MM samples. The expression of selected tRFs/tiRNAs were further validated in clinical specimens and myeloma cell lines by qPCR. Bioinformatic analysis was performed to predict their roles in multiple myeloma progression.We identified 10 upregulated tRFs/tiRNAs and 16 downregulated tRFs/tiRNAs. GO enrichment and KEGG pathway analysis were performed to analyse the functions of 1 significantly up-regulated and 1 significantly down-regulated tRNA-derived fragments. tRFs/tiRNAs may be involved in MM progression and drug-resistance.

**Conclusion:**

tRFs/tiRNAs were dysregulated and could be potential biomarkers for relapsed/refractory MM.

**Supplementary Information:**

The online version contains supplementary material available at 10.1186/s12859-021-04167-8.

## Introduction

Multiple myeloma (MM) is an incurable neoplastic plasma cell disorder, with a high tendency of relapse [1]. Although the advent of new drugs like proteasome inhibitor and CD38 monoclonal antibody have dramatically improved the prognoses, relapse and resistance to therapy frequently occur. The discovery of mechanisms of relapse and resistance is pivotal to optimizing the clinical efficacy and prolonging survival. Multiple factors have been proposed to be associated with relapsed/refractory MM (R/RMM). Of these, in vitro experiments have shown that Non-coding RNAs (ncRNAs) play important roles, but their clinical application value still need further exploration [2, 3].

NcRNAs are usually classified into long non-coding RNAs (lncRNAs) and small non-coding RNAs (sncRNAs) according to their length. Over past few decades, research has identified roles for ncRNAs in a variety of biological processes[4, 5]. With rapidly advances in high-throughput sequencing technology and bioinformatics analysis in recent years, a new class of sncRNAs derived from tRNAs are gaining increasing attention.

This new class of tRNA derived fragments can be broadly classified into tRNA related fragments (tRFs) and tRNA halves (tiRNAs). tRFs are generated from mature or precursor tRNA and tiRNAs are generated by specific cleavage in the anticodon loops of mature tRNA [6]. According to their mapped positions on the precursor or mature tRNA transcript, tRFs/tiRNAs are subdivided into five types: tRF-5, tRF-3, tRF-1, tRF-2 and tiRNA. tRFs/tiRNAs are involved in diverse molecular processes such as gene silencing, protein translation, cell stress, and cell differentiation [7, 8]. Though the complex biological functions of tRFs/tiRNAs require further elucidation, they can be summarized as three categories: translation regulation, epigenetic regulation and RNA silencing. These three categories have also been a key focus in cancer research of tRFs/tiRNAs in recent years.

Increasing evidence shows that tRFs/tiRNAs contribute to cancer development and progression. For example, tDR-0009 and tDR-7336, which were significantly upregulated in hypoxia conditions, have been found to induce doxorubicin resistance in triple-negative breast cancer [9]. In ovarian cancer, tRF-03357 was reported to promote cell proliferation, migration, and invasion by downregulating HMBOX1 [10]. For hematological malignancies, limited data showed that tRFs/tiRNAs may have cancer-associated functions in leukemia and lymphoma [11, 12]. And so far, there are no reports on the role of tRFs/tiRNAs in the mechanism of recurrence and drug resistance of MM to our knowledge.

In this study, we explored the expression profiles of tRFs/tiRNAs in new diagnosed MM(NDMM) and R/RMM samples by RNA-sequencing, with Quantitative Real-time PCR(qPCR)validation. Then, we analyzed their biological functions to uncover their roles in the mechanisms of relapse and drug resistance of MM. This study may provide potential biomarkers and therapeutic targets for R/RMM.

## Materials and methods

### Clinical specimens

Bone marrow specimens were obtained from patients with MM for research according to a protocol approved by ethics committee of the third Xiangya hospital. All patients provided their written informed consent to participate in this study.20 new diagnosed MM (NDMM) and 22 R/RMM patients were enrolled, respectively. All NDMM patients met the criteria for symptomatic multiple myeloma according to diagnostic criteria defined by National Comprehensive Cancer Network (NCCN). The diagnosis of relapsed/refractory disease was based on clinical symptoms, biochemical parameters and marrow evaluation. All R/RMM patients were in first relapse.

### Cell culture

MM cell lines U266 and RPMI-8226 were kindly provided by the basic laboratory of Central South University Xiangya School of Medicine. Drug-resistant cell lines U266/BTZ, and RPMI-8226/BTZ were induced through stepwise increase of drug concentrations. Cells were cultured in RPMI1640 (HyClone, Logan, UT, USA) supplemented with 10% FBS (ExCell Biology,Shanghai, China) and 1% penicillin–streptomycin (HyClone). All cells were incubated at 37 °C in 5% carbon dioxide.

### RNA Extraction and Quantitative RT-PCR

Firstly, anti-CD138 MicroBeads (Miltenyi, Germany) were used to enrich plasma cells from bone marrow samples by magnetic activated cell sorting (MACS). The purity of separated plasma cells was identified by flow cytometry and was all above 90%. Total RNA was then extracted from enriched plasma cells and myeloma cells using TRIzol (Invitrogen, USA) according to the instruction manual. Prepared RNA was stored at − 80 °C. RNA samples were qualified by agarose gel electrophoresis and quantified by NanoDrop ND-1000 (NanoDrop, USA). RNA integrity number (RIN) was evaluated by Agilent BioAnalyzer 2100 and was all above 7. RNA concentration and purity were also assessed (Additional file 1: Table 1). qRT-PCR was performed using ViiA 7 Real-time PCR System (Applied Biosystems) and 2 × PCR Master Mix. The expression levels of each tRFs/tiRNAs were calculated and normalized by U6 small nuclear RNA (snRNA). The primers used are listed in Table 1.

**Table 1:** Primers for qRT-PCR of candidate tRFs/tiRNAs

	Primer
tRF-60:77-Thr-TGT-1	F: 5′-AGCCATCCCAGTAGAGCCTC-3′ R: 5′-TATCCAGTGCAGGGTCCGAG-3′
tRF-1:22-Lys-TTT-1-M3	F: 5′-AGCCAGCCTGGATAGCTCAGT-3′ R: 5′-AGGGTCCGAGGTATTCGCA-3′
tRF- + 1:T17-Pro-TGG-3–2	F: 5′-TGGGTCGTGGCTACTGTTTT-3′ R:5′-AGTGCAGGGTCCGAGGTAT-3′
tRF-57:75-Gly-TCC-1-M3	F: 5′-TTATTCCCGGCCAACGCA-3′ R: 5′-CAGTGCAGGGTCCGAGGTAT-3′
tRF-1:31-Lys-CTT-1-M2	F: 5′-CTTGCCCGGCTAGCTCAGT-3′ R: 5′-CAGTGCAGGGTCCGAGGTAT-3′
tRF-1:32-Lys-CTT-1-M2	F: 5′-ATTGCCCGGCTAGCTCAGT-3′ R: 5′-CGCAGGGTCCGAGGTATTC-3′
U6	F: 5′-GCTTCGGCAGCACATATACTAAAAT-3′ R: 5′-CGCTTCACGAATTTGCGTGTCAT-3′

### Library preparation and sequencing

Before library preparation, total RNA samples from 5 NDMM and 5 R/RMM patients were pretreated to remove RNA modifications that interfere with small RNA-seq library construction. Then, cDNA was synthesized and amplified using Illumina’s proprietary RT primers and amplification primers. Subsequently, ~ 134 to 160 bp PCR amplified fragments were extracted and purified from the PAGE gel. Finally, the prepared libraries were quantified by Agilent BioAnalyzer 2100 and sequenced using Illumina NextSeq 500. Image analysis and base calling were performed by Solexa pipeline v1.8 (Off-Line Base Caller soft-ware, v1.8).

Sequencing quality were examined by FastQC and trimmed reads were aligned allowing for 1 mismatch only to the mature tRNA sequences. The abundance of tRFs/tiRNAs were evaluated using their sequencing counts and normalized as counts per million of total aligned reads (CPM). The differentially expressed tRFs/tiRNAs were calculated based on the count value with R package edgeR. Pie plots, Venn plots, Hierarchical clustering, Scatter plots and Volcano plots were constructed using R or Perl.

### Functional Analysis of tRFs/tiRNAs

Gene targets of tRFs/tiRNAs were predicted using TargetScan algorithms [13]. DAVID Bioinformatics Resources 6.8 was used for Gene Ontology (GO) and Kyoto Encyclopaedia of Genes and Genomes (KEGG) function enrichment analysis [14]. All data were graphed using Cytoscape 3.7.2 and GraphPad Prism 8.0.1.

### Statistical analysis

SPSS 23.0 software was used for statistical analysis. qPCR value was presented as mean ± standard deviation. KS test and Shapio-Wilk test were used to determine whether the data were normally distributed. For data that obeyed normal distribution, statistical significance was assessed using two-tail unpaired Student’s t test. Otherwise, non-parametric tests (Mann–Whitney test) were used for statistical analysis. *P* value < 0.05 was considered significant.

## Results

### Catalogue of tRFs/tiRNAs expression in MM

**Fig. 1:**
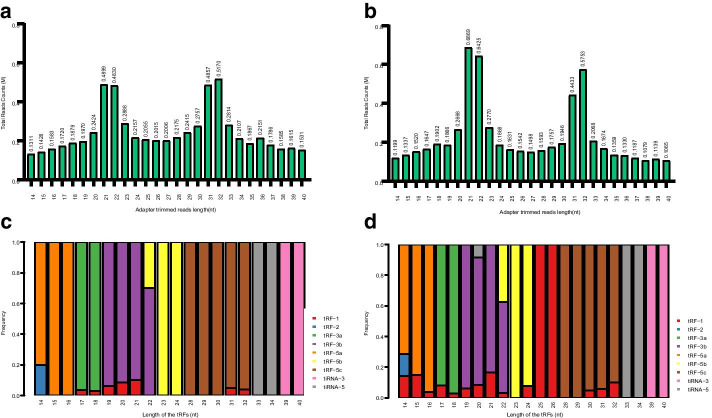
Catalogue of tRFs/tiRNAs expression profile between NDMM and R/RMM. **a** The average total read counts against the lengths of the trimmed reads in NDMM. **b** The average total read counts against the lengths of the trimmed reads in R/RMM. **c** The frequency of subtype against length of the tRFs/tiRNAs in NDMM. **d** The frequency of subtype against length of the tRFs/tiRNAs in R/RMM

After Illumina quality control and 5’, 3’-adaptor trimmed, reads with length < 14nt or length > 40nt were discarded. The bar diagrams show the sequence read length distribution (Fig. 1a, b). We also calculated the frequency of subtype tRFs/tiRNAs against the length of the sequence. The stacked bar charts show the length distribution of subtype (Fig. 1c, d). Clinical characteristics of all patients are shown in Table 2.

**Table 2:** baseline characteristics

	NDMM (n = 20)	R/RMM (n = 22)
*Age*		
Median—yr	59	60
Range—yr	38–75	44–76
*Sex-no. (%)*		
Male	11 (55.0)	14 (63.6)
Female	9 (45.0)	8 (36.4)
*ECOG performance status-no. (%)*		
0	7 (35.0)	6 (27.3)
1	11 (55.0)	13 (59.1)
2	2 (10.0)	3 (13.6)
*R-ISS stage-no. (%)*		
I	3 (15.0)	3 (13.6)
II	6 (30.0)	7 (31.8)
III	11 (55.0)	12 (54.5)
*Cytogenetics-no. (%)*		
High risk	6 (30.0)	10 (45.5)
Del (17p)	4 (20.0)	7 (31.8)
t (14;20)	2 (10.0)	3 (13.6)
t (14;16)	2 (10.0)	2 (9.1)
Standard risk	12 (60.0)	8 (36.3)
Unknown/missing	2 (10.0)	4 (18.2)
*No. of previous regimens-no. (%)*		
Median	–	3
Range	–	1–6
*Previous therapy-no. (%)*		
Bortezomib	–	18 (81.8)
Lenalidomide	–	8 (36.4)
ASCT	–	4 (18.2)

*ASCT* Autologous stem cell transplantation

### Differentially expressed tRFs/tiRNAs between NDMM and R/RMM groups

**Fig. 2:**
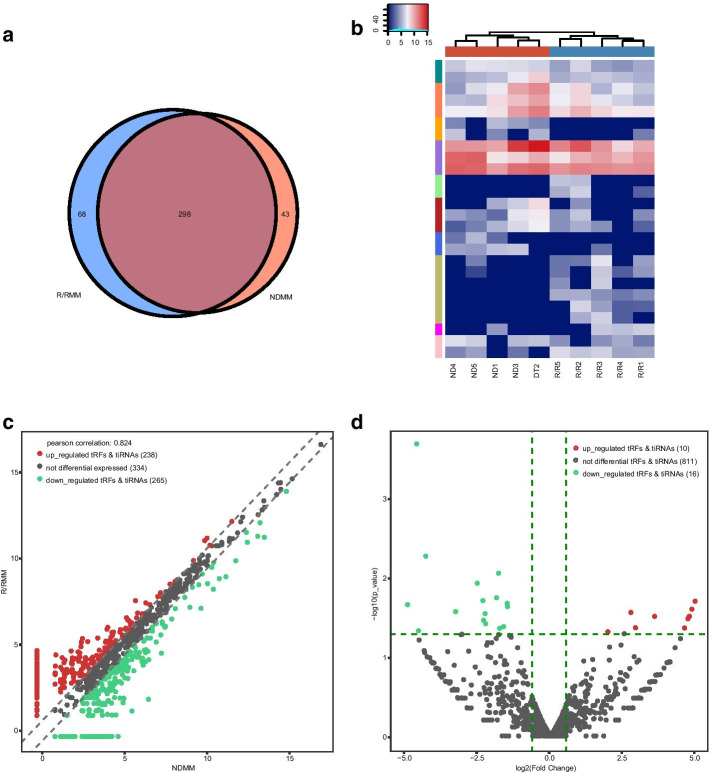
Expression profiles of tRFs/tiRNAs sequencing data in paired NDMM and R/RMM. **a** Venn diagram based on number of commonly expressed and specifically expressed tRFs/tiRNAs. **b** The hierarchical clustering heatmap for significantly differentially expressed tRFs/tiRNAs. **c** The scatter plot between two groups for tRFs/tiRNAs. **d** The volcano plot of tRFs/tiRNAs

Our high-throughput tRFs/tiRNAs sequencing identified more than 400 commonly expressed tRFs/tiRNAs (CPM ≥ 20). Of these, 298 tRFs/tiRNAs were commonly expressed in both groups, while 68 were specifically presented in R/RMM and 43 were specifically found in NDMM (specifically expressed tRFs/tiRNAs represent the CPM ≥ 20 in one group and < 20 in the other group) (Fig. 2a). Under the condition of fold change ≥ 1.5 and *P* < 0.05, of which 10 tRFs/tiRNAs were upregulated and 16 were downregulated. The heatmap shows hierarchical clustering of significantly differentially expressed tRFs/tiRNAs (Fig. 2b). The volcano and scatter plots indicate tRFs/tiRNAs expression variation between the two groups (Fig. 2c, d).

### tRF-60:77-Thr-TGT-1 was upregulated and tRF-1:22-Lys-TTT-1-M3 was downregulated in R/RMM and drug-resistant myeloma cells

**Fig. 3:**
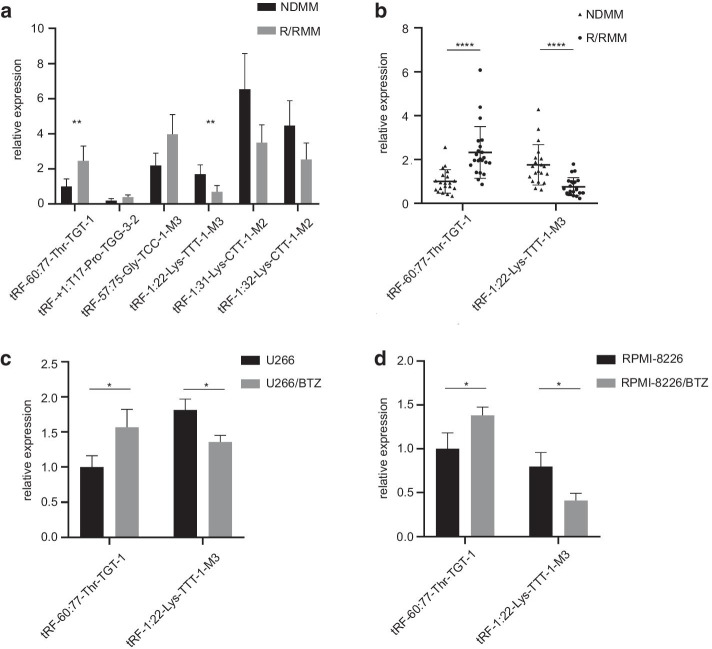
Relative expression of tRFs/tiRNAs by qPCR. **a** Relative expression of 3 significantly upregulated or downregulated tRFs/tiRNAs in paired NDMM and R/RMM samples. **b** Relative expression of tRF-60:77-Thr-TGT-1 and tRF-1:22-Lys-TTT-1-M3 in 22 R/RMM and 20 NDMM patients. **c** Relative expression of tRF-60:77-Thr-TGT-1 and tRF-1:22-Lys-TTT-1-M3 in U266 and BTZ-resistant U266. **d** Relative expression of tRF-60:77-Thr-TGT-1 and tRF-1:22-Lys-TTT-1-M3 in RPMI-8266 and BTZ-resistant RPMI-8266

qPCR was performed to confirm the expression of three significantly upregulated or downregulated tRFs/tiRNAs in NDMM and R/RMM samples. The expression of all these six tRFs/tiRNAs were consistent with the sequencing data, while the most significantly upregulated and downregulated tRFs/tiRNAs were tRF-60:77-Thr-TGT-1 and tRF-1:22-Lys-TTT-1-M3, respectively (Fig. 3a). For the further validation, we detected expression of tRF-60:77-Thr-TGT-1 and tRF-1:22-Lys-TTT-1-M3 in 22 R/RMM and 20 NDMM patients (Fig. 3b). Their expression was also detected in MM cell lines (U266, RPMI-8226) and drug-resistant cell lines (U266/BTZ, RPMI-8226/BTZ). tRF-60:77-Thr-TGT-1 was upregulated and tRF-1:22-Lys-TTT-1-M3 was downregulated in both R/RMM and drug-resistant myeloma cells (Fig. 3c, d). There seems to be no significant difference in the expression of tRF-60:77-Thr-TGT-1 or tRF-1:22-Lys-TTT-1-M3 among patients with different cytogenetic risk in NDMM or R/RMM group. Which may be related to the relatively small sample size. Absolute values of RNA expression levels are shown in Additional file 2: Table 2.

### Target gene prediction with bioinformatics tool

**Fig. 4:**
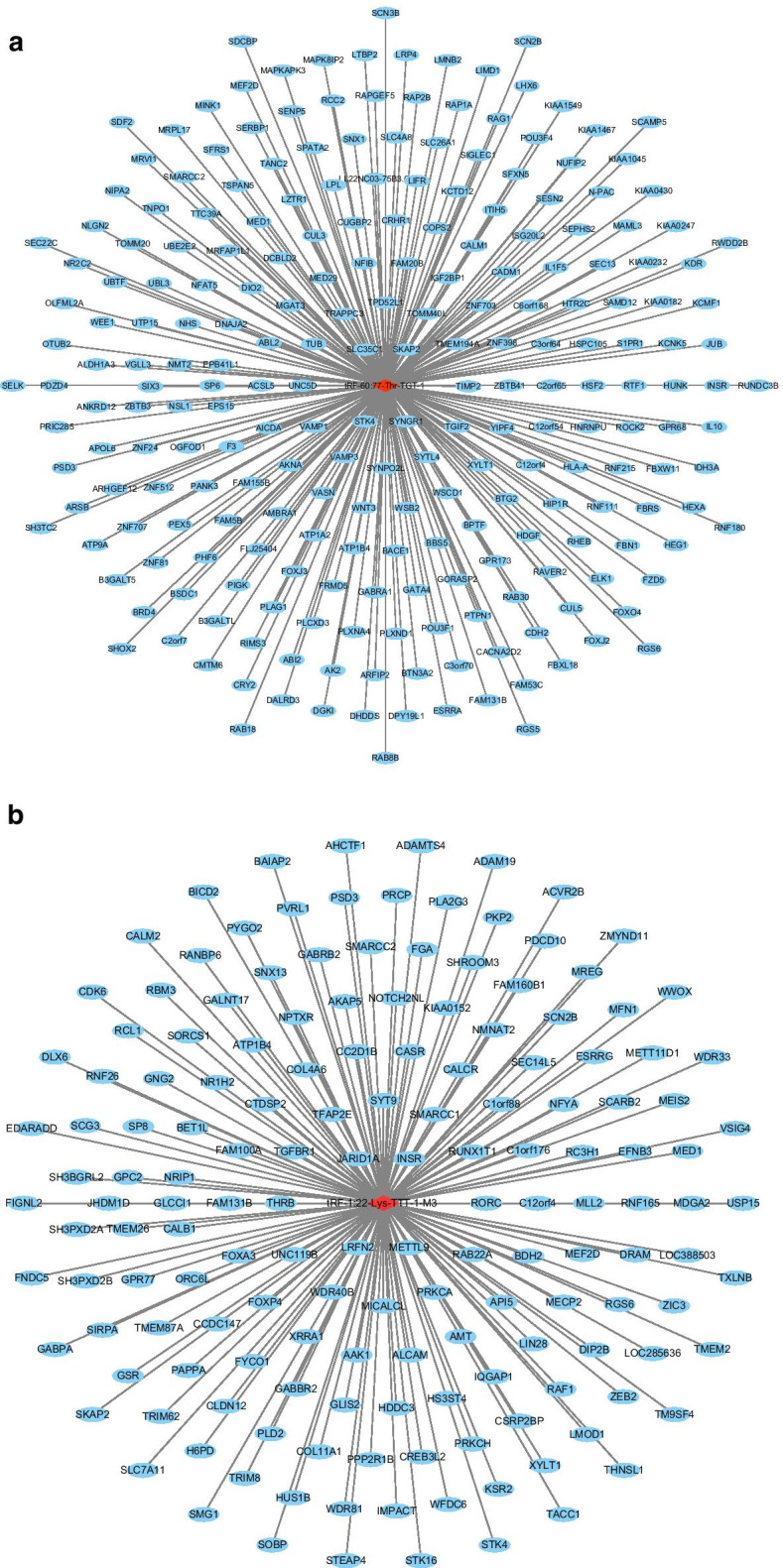
Target gene prediction of tRFs/tiRNAs. **a** Target genes of tRF-60:77-Thr-TGT-1. **b** Target genes of tRF-1:22-Lys-TTT-1-M3

TargetScan algorithms were used to explore the putative roles of tRF-60:77-Thr-TGT-1 and tRF-1:22-Lys-TTT-1-M3 in MM. Through this strategy, we predicted 238 conserved targets and 159 conserved targets, respectively. The network diagrams constructed by Cytoscape show the genes (Fig. 4a, b).

### GO enrichment analysis and KEGG pathway analysis

**Fig. 5:**
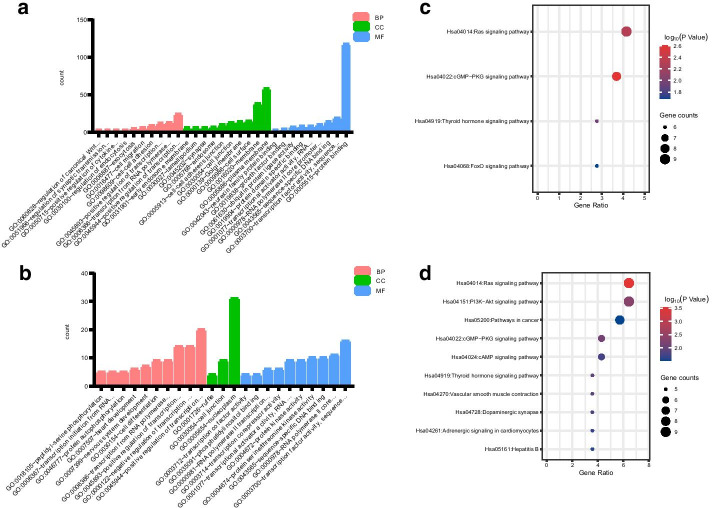
Functional analysis of tRFs/tiRNAs. **a** GO enrichment analysis of tRF-60:77-Thr-TGT-1. **b** GO enrichment analysis of tRF-1:22-Lys-TTT-1-M3. **c** KEGG pathway analysis of tRF-60:77-Thr-TGT-1. **d** KEGG pathway analysis of tRF-1:22-Lys-TTT-1-M3

We performed functional enrichment analysis for target genes of tRF-60:77-Thr-TGT-1 and tRF-1:22-Lys-TTT-1-M3 through DAVID database. GO analysis indicated that 37 GO terms were enriched (*P* < 0.05) for both target genes of tRF-60:77-Thr-TGT-1 and tRF-1:22-Lys-TTT-1-M3. The most enriched terms of target genes of tRF-60:77-Thr-TGT-1 were ‘plasma membrane’ and ‘membrane’ in cellular component (CC) category, ‘protein binding’ and ‘transcription factor activity, sequence-specific DNA binding’ in molecular function (MF) category, ‘positive regulation of transcription from RNA polymerase II promoter’ and ‘transcription from RNA polymerase II promoter’ in biological process (BP) category (Fig. 5a). Whereas, the most enriched terms of target genes of tRF-1:22-Lys-TTT-1-M3 were ‘nucleoplasm’ and ‘cell junction’ in CC category, ‘transcription factor activity, sequence-specific DNA binding’ and ‘RNA polymerase II core promoter proximal region sequence-specific DNA binding’ in MF category, ‘positive regulation of transcription, DNA-templated’ and ‘positive regulation of transcription from RNA polymerase II promoter’ in BP category (Fig. 5b).

KEGG pathway analysis showed that targets of tRF-60:77-Thr-TGT-1 might participate in Ras signaling pathway, cGMP-PKG signaling pathway, thyroid hormone signaling pathway and FoxO signaling pathway (*P* < 0.05) (Fig. 5c). For targets of tRF-1:22-Lys-TTT-1-M3, the most enriched pathways were Ras signaling pathway, PI3K-Akt signaling pathway and pathways in cancer (Fig. 5d).

## Discussion

MM is an incurable hematological malignancy. Even patients who are initially sensitive to treatment will eventually develop relapsed and refractory disease. So far, many genes or ncRNAs related to relapse and drug resistance of MM have been identified. For example, J Xia demonstrated that NEK2 induces autophagy-mediated bortezomib resistance by stabilizing Beclin-1 in multiple myeloma [15]. MicroRNA-497 was found to inhibit myeloma growth and increase susceptibility to bortezomib by targeting Bcl-2 [16]. B-Z Zhu reported that LncRNA HOTAIR activated the expression of NF-κB and promoted the proliferation of myeloma cells [17]. However, it is still unclear whether tRFs/tiRNAs are involved in the relapse and drug resistance of MM. Therefore, for the first time, we conducted a preliminary evaluation of the role of tRFs/tiRNAs in R/RMM

We used the standardized tDR naming system to name the tRFs/tiRNAs involved in this study [18]. The 4 parts in the name represent prefix, position, source tRNA and matching tRNA transcripts in turn. Two potentially significant tRFs were finally screened out by RNA sequencing and qPCR validation. To explore the pathological processes that they may be involved in, we conducted functional analysis of the predicted target genes. For tRF-60:77-Thr-TGT-1, its predicted target genes were mainly located on membrane and implicated in transcription initiation, cell adhesion and migration. Whereas, target genes of tRF-1:22-Lys-TTT-1-M3 were primarily localized in nucleoplasm. Similarly, they principally regulated transcription initiation by exerting transcription factor activity. This is consistent with the three major functional categories of tRFs/tiRNAs summarized in the previous section. The mechanism of tRFs/tiRNAs-mediated translation regulation is complicated. For example, it was reported that 5’-tiRNA-associated translational silencer Y-Box-Binding Protein 1(YB-1) could contribute to stress-induced translational repression [19]. A universal conserved ‘GG’ di-nucleotide in 5’-tRFs was also involved in the process of protein translation [20]. Moreover, tRFs/tiRNAs could regulate translation by interacting with ribosomes [21, 22]. The transcriptional regulation mechanism of tRFs/tiRNAs in R/RMM needs further investigation.

We noticed that both tRFs were involved in the Ras signaling pathway in KEGG pathway analysis. The Ras pathway has been found to regulate many fundamental biological processes, like cell proliferation, differentiation, survival, and apoptosis. Dysregulation of this pathway generate the emergence, development, and progression of multiple cancers [23–25]. In R/RMM, Ras mutations may increase from 23–54 to 45–81% compared with NDMM [26, 27]. The Ras/ Raf/MEK/Erk pathway is associated with drug resistance and a more aggressive phenotype in MM [28]. In addition, these differentially expressed tRFs/tiRNAs may also participate in a variety of cancer-related signaling pathways such as FoxO signaling pathway, PI3K-Akt-mTOR signaling pathway. FOXO3a transcription factor can be negatively regulated by mTOR complex 2(mTORC2) and then induces a pro-survival response, which suggests potential new mechanism of inhibition of mTORC2 in MM [29]. Sorafenib, an orally compound that acts predominantly by inhibition of Raf kinase and VEGF receptor 2 can inhibit the proliferation of MM cells. It has also been tested in combination with rapamycin to inhibit mTOR, and this shows synergistic effects on MM cells in vitro [30]. In short, these provide reference for the design of more precision and individualized clinical trials. To date, both up-regulated or down-regulated expression tRFs/tiRNAs has been reported to play a pro-tumor role in tumor tissues [10, 31]. Therefore, additional experiments need to be conducted to validate these hypotheses.

There remain several limitations in this study. For example, tRFs/tiRNAs can also perform their biological functions through regulating miRNA activity [32]. Regrettably, current bioinformatics methods are not able to predict the target miRNAs of tRFs/tiRNAs. Therefore, we failed to get the ceRNA network. We plan to explore how tRFs/tiRNAs regulate miRNA in MM in future molecular biology experiments. Besides, according to serum M protein, myeloma can be categorized into eight subclasses (type IgG, type IgA, type IgD, type IgE, type IgM, light chain type, non-secreted type and polyclonal type). Different types of MM may have different phenotypes. In this study, we used two cell lines (IgE or IgG secreting) for in vitro verification. Although this is the practice in most myeloma-related studies, but additional more cell lines would be beneficial to confirm the data.

In summary, we evaluated the potential function of tRFs/tiRNAs in R/RMM and the results provide the basis for preclinical research. Studies on tRFs/tiRNAs are still in their infancy and deep insights into their roles in carcinogenesis and progression remain lacking. tRFs/tiRNAs have the potential as clinical biomarkers for drug resistance and prognosis of MM and may be used as treatment targets in the future.

## Supplementary Information

**Additional file 1**. RNA quality Quantification and Quality Assurance.

**Additional file 2**. Absolute values of RNA expression (×10^−3^).

## Data Availability

All data generated or analyzed during this study are included in this published article. Sequencing data has been uploaded to GEO database (available at https://www.ncbi.nlm.nih.gov/geo/query/acc.cgi?acc=GSE171003). The accession number is GSE171003. We are currently studying the mechanism of these tRFs/tiRNAs. In order to ensure the novelty of key molecules, we have not provide the sequencing data publicly for the time being.
