# Supplementary material for: tRNA-derived fragments as novel potential biomarkers for relapsed/refractory multiple myeloma
Source: BMC Bioinformatics. 2021 May 11;22:238. doi: 10.1186/s12859-021-04167-8 (PMC8111751; doi:10.1186/s12859-021-04167-8)
Supplement: Supplementary file 1 — Additional file 1. RNA quality Quantification and Quality Assurance. [file 12859_2021_4167_MOESM1_ESM.docx]

Supplementary table 1. RNA quality Quantification and Quality Assurance.

| Sample ID | OD 260/280 Ratio | OD 260/230 Ratio | Conc. (ng/μl) | Volume (μl) |
| --- | --- | --- | --- | --- |
| NDMM 01  NDMM 02  NDMM 03  NDMM 04  NDMM 05  NDMM 06  NDMM 07  NDMM 08  NDMM 09  NDMM 10  NDMM 11  NDMM 12  NDMM 13  NDMM 14  NDMM 15  NDMM 16  NDMM 17  NDMM 18  NDMM 19  NDMM 20  R/RMM 01  R/RMM 02  R/RMM 03  R/RMM 04  R/RMM 05  R/RMM 06  R/RMM 07  R/RMM 08  R/RMM 09  R/RMM 10  R/RMM 11  R/RMM 12  R/RMM 13  R/RMM 14  R/RMM 15  R/RMM 16  R/RMM 17  R/RMM 18  R/RMM 19  R/RMM 20  R/RMM 21  R/RMM 22 | 1.86  1.79  1.76  1.92  1.95  1.88  1.77  1.90  1.86  1.79  1.89  1.92  1.78  1.85  1.88  1.84  1.83  1.90  1.83  1.86  1.85  1.91  1.76  1.81  1.75  1.94  1.88  1.83  1.79  1.86  1.82  1.84  1.89  1.91  1.84  1.86  1.90  1.82  1.75  1.92  1.84  1.86 | 1.77  1.89  1.84  1.87  1.85  1.94  1.90  1.88  1.85  1.93  1.98  1.80  1.95  1.92  1.79  1.82  1.87  1.93  1.97  1.94  1.91  1.82  1.92  1.87  1.88  1.83  1.86  1.93  1.99  1.91  1.87  1.83  1.89  1.93  1.95  1.89  1.92  1.84  1.93  1.96  1.82  1.87 | 133.47  103.72  158.18  97.73  154.90  131.89  74.75  218.03  130.39  238.94  152.68  65.53  338.54  316.43  167.86  186.67  195.47  247.56  148.54  256.87  156.77  68.83  307.41  178.56  233.73  185.37  277.94  148.41  389.93  137.84  253.47  144.11  189.34  67.36  153.75  311.37  265.58  238.26  132.98  178.36  308.99  88.43 | 30  30  30  30  30  30  30  30  30  30  30  30  30  30  30  30  30  30  30  30  30  30  30  30  30  30  30  30  30  30  30  30  30  30  30  30  30  30  30  30  30  30 |
